# Supplementary material for: Role and mechanism of NCAPD3 in promoting malignant behaviors in gastric cancer
Source: Front Pharmacol. 2024 Apr 22;15:1341039. doi: 10.3389/fphar.2024.1341039 (PMC11070777; doi:10.3389/fphar.2024.1341039)
Supplement: Supplementary file 11 [file DataSheet2.ZIP › GSEA/Transcipt factor/my_analysis.Gsea.1599461814734/gsea_report_for_WT_1599461814734.html]

Report for WT 1599461814734 [GSEA]

| GS  follow link to MSigDB | GS DETAILS | SIZE | ES | NES | NOM p-val | FDR q-val | FWER p-val | RANK AT MAX | LEADING EDGE || 1 | DIDO1\_TARGET\_GENES | Details ... | 146 | 0.31 | 2.31 | 0.000 | 0.046 | 0.056 | 698 | tags=73%, list=49%, signal=129% |
| 2 | SETD1A\_TARGET\_GENES | Details ... | 134 | 0.28 | 2.02 | 0.000 | 0.319 | 0.545 | 631 | tags=65%, list=45%, signal=106% |
| 3 | SUPT20H\_TARGET\_GENES | Details ... | 119 | 0.27 | 1.99 | 0.000 | 0.277 | 0.660 | 698 | tags=72%, list=49%, signal=131% |
| 4 | AR\_Q6 | Details ... | 18 | 0.49 | 1.99 | 0.007 | 0.212 | 0.663 | 495 | tags=72%, list=35%, signal=110% |
| 5 | ADA2\_TARGET\_GENES | Details ... | 79 | 0.31 | 1.98 | 0.000 | 0.181 | 0.687 | 700 | tags=76%, list=50%, signal=142% |
| 6 | ARID5B\_TARGET\_GENES | Details ... | 95 | 0.29 | 1.96 | 0.000 | 0.169 | 0.724 | 695 | tags=71%, list=49%, signal=130% |
| 7 | THAP1\_TARGET\_GENES | Details ... | 161 | 0.24 | 1.89 | 0.002 | 0.265 | 0.904 | 735 | tags=71%, list=52%, signal=132% |
| 8 | ZNF711\_TARGET\_GENES | Details ... | 150 | 0.24 | 1.85 | 0.003 | 0.286 | 0.945 | 759 | tags=72%, list=54%, signal=139% |
| 9 | ELF2\_TARGET\_GENES | Details ... | 74 | 0.29 | 1.82 | 0.002 | 0.314 | 0.969 | 509 | tags=58%, list=36%, signal=86% |
| 10 | DLX4\_TARGET\_GENES | Details ... | 91 | 0.27 | 1.81 | 0.002 | 0.314 | 0.976 | 696 | tags=71%, list=49%, signal=132% |
| 11 | SCGGAAGY\_ELK1\_02 | Details ... | 126 | 0.24 | 1.79 | 0.000 | 0.324 | 0.983 | 720 | tags=71%, list=51%, signal=131% |
| 12 | NRF1\_Q6 | Details ... | 23 | 0.38 | 1.71 | 0.029 | 0.489 | 0.999 | 307 | tags=43%, list=22%, signal=55% |
| 13 | HOXA1\_TARGET\_GENES | Details ... | 121 | 0.23 | 1.70 | 0.010 | 0.494 | 1.000 | 491 | tags=48%, list=35%, signal=67% |
| 14 | ALKBH3\_TARGET\_GENES | Details ... | 37 | 0.33 | 1.70 | 0.020 | 0.474 | 1.000 | 735 | tags=81%, list=52%, signal=165% |
| 15 | IRF5\_TARGET\_GENES | Details ... | 67 | 0.27 | 1.66 | 0.012 | 0.541 | 1.000 | 416 | tags=45%, list=29%, signal=60% |
| 16 | ZSCAN5DP\_TARGET\_GENES | Details ... | 160 | 0.22 | 1.65 | 0.010 | 0.545 | 1.000 | 733 | tags=67%, list=52%, signal=123% |
| 17 | ZSCAN31\_TARGET\_GENES | Details ... | 84 | 0.25 | 1.65 | 0.008 | 0.515 | 1.000 | 196 | tags=27%, list=14%, signal=30% |
| 18 | NFMUE1\_Q6 | Details ... | 29 | 0.34 | 1.64 | 0.020 | 0.512 | 1.000 | 552 | tags=62%, list=39%, signal=100% |
| 19 | TCCCRNNRTGC\_UNKNOWN | Details ... | 23 | 0.36 | 1.62 | 0.024 | 0.548 | 1.000 | 547 | tags=65%, list=39%, signal=105% |
| 20 | ZNF407\_TARGET\_GENES | Details ... | 157 | 0.22 | 1.61 | 0.004 | 0.562 | 1.000 | 553 | tags=51%, list=39%, signal=74% |
| 21 | MTA1\_TARGET\_GENES |  | 191 | 0.20 | 1.61 | 0.012 | 0.546 | 1.000 | 630 | tags=57%, list=45%, signal=88% |
| 22 | NR1D1\_TARGET\_GENES |  | 17 | 0.41 | 1.60 | 0.050 | 0.529 | 1.000 | 569 | tags=76%, list=40%, signal=127% |
| 23 | CREB\_02 |  | 30 | 0.33 | 1.60 | 0.036 | 0.532 | 1.000 | 243 | tags=37%, list=17%, signal=43% |
| 24 | ZNF146\_TARGET\_GENES |  | 80 | 0.24 | 1.58 | 0.024 | 0.550 | 1.000 | 644 | tags=63%, list=46%, signal=108% |
| 25 | SREBP1\_Q6 |  | 26 | 0.34 | 1.57 | 0.033 | 0.559 | 1.000 | 337 | tags=42%, list=24%, signal=55% |
| 26 | HES4\_TARGET\_GENES |  | 55 | 0.27 | 1.56 | 0.057 | 0.574 | 1.000 | 569 | tags=58%, list=40%, signal=94% |
| 27 | PBX1\_01 |  | 15 | 0.40 | 1.56 | 0.051 | 0.569 | 1.000 | 165 | tags=33%, list=12%, signal=37% |
| 28 | GFI1\_01 |  | 21 | 0.36 | 1.55 | 0.068 | 0.573 | 1.000 | 287 | tags=48%, list=20%, signal=59% |
| 29 | STAT1\_02 |  | 22 | 0.36 | 1.54 | 0.059 | 0.588 | 1.000 | 104 | tags=27%, list=7%, signal=29% |
| 30 | E2F\_Q3\_01 |  | 28 | 0.32 | 1.53 | 0.049 | 0.600 | 1.000 | 688 | tags=75%, list=49%, signal=143% |
| 31 | ZNF260\_TARGET\_GENES |  | 70 | 0.24 | 1.53 | 0.054 | 0.584 | 1.000 | 374 | tags=40%, list=27%, signal=52% |
| 32 | TAF9B\_TARGET\_GENES |  | 54 | 0.26 | 1.51 | 0.041 | 0.611 | 1.000 | 661 | tags=67%, list=47%, signal=121% |
| 33 | GLI4\_TARGET\_GENES |  | 70 | 0.24 | 1.51 | 0.050 | 0.594 | 1.000 | 457 | tags=46%, list=32%, signal=64% |
| 34 | MYB\_Q6 |  | 22 | 0.34 | 1.49 | 0.061 | 0.633 | 1.000 | 702 | tags=77%, list=50%, signal=151% |
| 35 | YY1\_01 |  | 26 | 0.31 | 1.49 | 0.073 | 0.622 | 1.000 | 159 | tags=27%, list=11%, signal=30% |
| 36 | NKX2\_5\_TARGET\_GENES |  | 127 | 0.20 | 1.48 | 0.031 | 0.629 | 1.000 | 506 | tags=49%, list=36%, signal=69% |
| 37 | HIVEP1\_TARGET\_GENES |  | 19 | 0.35 | 1.45 | 0.081 | 0.713 | 1.000 | 361 | tags=53%, list=26%, signal=70% |
| 38 | HOXB6\_TARGET\_GENES |  | 51 | 0.25 | 1.45 | 0.067 | 0.699 | 1.000 | 447 | tags=47%, list=32%, signal=66% |
| 39 | MYBL1\_TARGET\_GENES |  | 23 | 0.33 | 1.45 | 0.098 | 0.683 | 1.000 | 644 | tags=74%, list=46%, signal=134% |
| 40 | FOXN3\_TARGET\_GENES |  | 113 | 0.20 | 1.44 | 0.048 | 0.707 | 1.000 | 253 | tags=26%, list=18%, signal=29% |
| 41 | TGCGCANK\_UNKNOWN |  | 56 | 0.24 | 1.44 | 0.082 | 0.711 | 1.000 | 521 | tags=52%, list=37%, signal=79% |
| 42 | AC118549\_1\_TARGET\_GENES |  | 31 | 0.28 | 1.42 | 0.101 | 0.756 | 1.000 | 604 | tags=65%, list=43%, signal=110% |
| 43 | E2F1\_Q4\_01 |  | 29 | 0.29 | 1.41 | 0.096 | 0.756 | 1.000 | 688 | tags=72%, list=49%, signal=138% |
| 44 | ZSCAN2\_TARGET\_GENES |  | 76 | 0.22 | 1.41 | 0.067 | 0.752 | 1.000 | 379 | tags=37%, list=27%, signal=48% |
| 45 | ZNF524\_TARGET\_GENES |  | 27 | 0.30 | 1.41 | 0.101 | 0.736 | 1.000 | 417 | tags=48%, list=30%, signal=67% |
| 46 | AAAYRNCTG\_UNKNOWN |  | 27 | 0.30 | 1.41 | 0.102 | 0.732 | 1.000 | 352 | tags=44%, list=25%, signal=58% |
| 47 | ZNF354B\_TARGET\_GENES |  | 28 | 0.30 | 1.40 | 0.095 | 0.749 | 1.000 | 829 | tags=93%, list=59%, signal=221% |
| 48 | XBP1\_01 |  | 18 | 0.34 | 1.37 | 0.125 | 0.837 | 1.000 | 547 | tags=67%, list=39%, signal=107% |
| 49 | ZNF581\_TARGET\_GENES |  | 39 | 0.26 | 1.36 | 0.110 | 0.852 | 1.000 | 267 | tags=33%, list=19%, signal=40% |
| 50 | AUTS2\_TARGET\_GENES |  | 60 | 0.23 | 1.36 | 0.098 | 0.836 | 1.000 | 360 | tags=35%, list=26%, signal=45% |
| 51 | U2AF2\_TARGET\_GENES |  | 82 | 0.21 | 1.36 | 0.104 | 0.825 | 1.000 | 649 | tags=61%, list=46%, signal=106% |
| 52 | ZNF561\_TARGET\_GENES |  | 77 | 0.21 | 1.36 | 0.117 | 0.820 | 1.000 | 461 | tags=43%, list=33%, signal=60% |
| 53 | STAT5A\_04 |  | 17 | 0.34 | 1.35 | 0.126 | 0.817 | 1.000 | 54 | tags=24%, list=4%, signal=24% |
| 54 | ZNF784\_TARGET\_GENES |  | 41 | 0.25 | 1.35 | 0.122 | 0.835 | 1.000 | 427 | tags=44%, list=30%, signal=61% |
| 55 | ZNF85\_TARGET\_GENES |  | 24 | 0.30 | 1.34 | 0.151 | 0.843 | 1.000 | 145 | tags=25%, list=10%, signal=27% |
| 56 | ETS2\_TARGET\_GENES |  | 46 | 0.24 | 1.34 | 0.135 | 0.846 | 1.000 | 257 | tags=28%, list=18%, signal=33% |
| 57 | MTF1\_Q4 |  | 20 | 0.31 | 1.33 | 0.161 | 0.846 | 1.000 | 326 | tags=40%, list=23%, signal=51% |
| 58 | E2F1\_Q6\_01 |  | 33 | 0.26 | 1.33 | 0.145 | 0.835 | 1.000 | 680 | tags=70%, list=48%, signal=131% |
| 59 | TET1\_TARGET\_GENES |  | 111 | 0.19 | 1.32 | 0.110 | 0.848 | 1.000 | 410 | tags=38%, list=29%, signal=49% |
| 60 | GRHL1\_TARGET\_GENES |  | 162 | 0.17 | 1.32 | 0.111 | 0.844 | 1.000 | 609 | tags=52%, list=43%, signal=81% |
| 61 | ZNF205\_TARGET\_GENES |  | 168 | 0.17 | 1.31 | 0.122 | 0.863 | 1.000 | 306 | tags=27%, list=22%, signal=31% |
| 62 | ETS1\_B |  | 22 | 0.31 | 1.31 | 0.162 | 0.856 | 1.000 | 63 | tags=23%, list=4%, signal=23% |
| 63 | OVOL3\_TARGET\_GENES |  | 45 | 0.24 | 1.30 | 0.144 | 0.867 | 1.000 | 550 | tags=56%, list=39%, signal=88% |
| 64 | DOT1L\_TARGET\_GENES |  | 37 | 0.25 | 1.30 | 0.172 | 0.864 | 1.000 | 517 | tags=54%, list=37%, signal=83% |
| 65 | MEF2C\_TARGET\_GENES |  | 141 | 0.18 | 1.30 | 0.124 | 0.853 | 1.000 | 424 | tags=36%, list=30%, signal=47% |
| 66 | PTPRA\_TARGET\_GENES |  | 15 | 0.33 | 1.30 | 0.158 | 0.848 | 1.000 | 710 | tags=80%, list=50%, signal=159% |
| 67 | SRPK1\_TARGET\_GENES |  | 28 | 0.26 | 1.30 | 0.174 | 0.838 | 1.000 | 577 | tags=61%, list=41%, signal=101% |
| 68 | NRF2\_01 |  | 33 | 0.27 | 1.30 | 0.184 | 0.828 | 1.000 | 595 | tags=61%, list=42%, signal=102% |
| 69 | AACWWCAANK\_UNKNOWN |  | 19 | 0.31 | 1.30 | 0.163 | 0.821 | 1.000 | 488 | tags=53%, list=35%, signal=79% |
| 70 | CHAMP1\_TARGET\_GENES |  | 20 | 0.31 | 1.29 | 0.150 | 0.811 | 1.000 | 672 | tags=75%, list=48%, signal=141% |
| 71 | DBP\_TARGET\_GENES |  | 145 | 0.17 | 1.29 | 0.131 | 0.802 | 1.000 | 484 | tags=43%, list=34%, signal=58% |
| 72 | SMTTTTGT\_UNKNOWN |  | 51 | 0.22 | 1.29 | 0.155 | 0.816 | 1.000 | 142 | tags=20%, list=10%, signal=21% |
| 73 | PR\_Q2 |  | 15 | 0.33 | 1.28 | 0.191 | 0.836 | 1.000 | 237 | tags=40%, list=17%, signal=48% |
| 74 | NFE2L1\_TARGET\_GENES |  | 43 | 0.23 | 1.28 | 0.169 | 0.826 | 1.000 | 239 | tags=26%, list=17%, signal=30% |
| 75 | BARX2\_TARGET\_GENES |  | 164 | 0.16 | 1.27 | 0.146 | 0.835 | 1.000 | 244 | tags=23%, list=17%, signal=24% |
| 76 | GCCATNTTG\_YY1\_Q6 |  | 49 | 0.22 | 1.27 | 0.173 | 0.833 | 1.000 | 848 | tags=82%, list=60%, signal=197% |
| 77 | IK3\_01 |  | 15 | 0.33 | 1.26 | 0.205 | 0.845 | 1.000 | 70 | tags=27%, list=5%, signal=28% |
| 78 | MCM3\_TARGET\_GENES |  | 24 | 0.27 | 1.24 | 0.210 | 0.906 | 1.000 | 735 | tags=75%, list=52%, signal=154% |
| 79 | CIITA\_TARGET\_GENES |  | 126 | 0.17 | 1.24 | 0.161 | 0.898 | 1.000 | 720 | tags=63%, list=51%, signal=117% |
| 80 | E2F\_Q4\_01 |  | 31 | 0.25 | 1.23 | 0.210 | 0.934 | 1.000 | 688 | tags=68%, list=49%, signal=129% |
| 81 | HOXB4\_TARGET\_GENES |  | 101 | 0.18 | 1.23 | 0.174 | 0.929 | 1.000 | 287 | tags=29%, list=20%, signal=33% |
| 82 | KMT2D\_TARGET\_GENES |  | 71 | 0.20 | 1.23 | 0.208 | 0.921 | 1.000 | 82 | tags=15%, list=6%, signal=16% |
| 83 | CMYB\_01 |  | 24 | 0.26 | 1.22 | 0.239 | 0.924 | 1.000 | 563 | tags=58%, list=40%, signal=95% |
| 84 | YGCANTGCR\_UNKNOWN |  | 16 | 0.31 | 1.22 | 0.222 | 0.919 | 1.000 | 219 | tags=31%, list=16%, signal=37% |
| 85 | YBX3\_TARGET\_GENES |  | 28 | 0.25 | 1.22 | 0.229 | 0.911 | 1.000 | 630 | tags=68%, list=45%, signal=120% |
| 86 | TGCTGAY\_UNKNOWN |  | 32 | 0.24 | 1.22 | 0.227 | 0.906 | 1.000 | 181 | tags=25%, list=13%, signal=28% |
| 87 | ZBED5\_TARGET\_GENES |  | 145 | 0.16 | 1.22 | 0.199 | 0.899 | 1.000 | 516 | tags=42%, list=37%, signal=60% |
| 88 | NFKBIA\_TARGET\_GENES |  | 75 | 0.19 | 1.21 | 0.230 | 0.917 | 1.000 | 303 | tags=29%, list=21%, signal=35% |
| 89 | ZNF257\_TARGET\_GENES |  | 40 | 0.22 | 1.20 | 0.234 | 0.932 | 1.000 | 287 | tags=30%, list=20%, signal=37% |
| 90 | ZNF22\_TARGET\_GENES |  | 96 | 0.18 | 1.20 | 0.216 | 0.923 | 1.000 | 419 | tags=36%, list=30%, signal=48% |
| 91 | ZBTB18\_TARGET\_GENES |  | 72 | 0.19 | 1.20 | 0.220 | 0.914 | 1.000 | 769 | tags=71%, list=55%, signal=148% |
| 92 | AP4\_Q5 |  | 20 | 0.28 | 1.20 | 0.251 | 0.910 | 1.000 | 46 | tags=20%, list=3%, signal=20% |
| 93 | TAXCREB\_01 |  | 17 | 0.30 | 1.20 | 0.260 | 0.906 | 1.000 | 28 | tags=18%, list=2%, signal=18% |
| 94 | GABP\_B |  | 35 | 0.24 | 1.20 | 0.250 | 0.903 | 1.000 | 711 | tags=71%, list=50%, signal=140% |
| 95 | MYOGENIN\_Q6 |  | 16 | 0.30 | 1.18 | 0.255 | 0.945 | 1.000 | 37 | tags=19%, list=3%, signal=19% |
| 96 | ER\_Q6 |  | 26 | 0.25 | 1.18 | 0.243 | 0.941 | 1.000 | 93 | tags=19%, list=7%, signal=20% |
| 97 | SMN1\_TARGET\_GENES |  | 60 | 0.20 | 1.18 | 0.233 | 0.937 | 1.000 | 629 | tags=55%, list=45%, signal=95% |
| 98 | BARHL1\_TARGET\_GENES |  | 115 | 0.16 | 1.18 | 0.242 | 0.930 | 1.000 | 639 | tags=56%, list=45%, signal=93% |
| 99 | SMN2\_TARGET\_GENES |  | 60 | 0.20 | 1.17 | 0.238 | 0.932 | 1.000 | 629 | tags=55%, list=45%, signal=95% |
| 100 | ZNF213\_TARGET\_GENES |  | 53 | 0.20 | 1.17 | 0.256 | 0.924 | 1.000 | 427 | tags=40%, list=30%, signal=55% |
| 101 | TGGAAA\_NFAT\_Q4\_01 |  | 163 | 0.15 | 1.17 | 0.233 | 0.925 | 1.000 | 98 | tags=13%, list=7%, signal=12% |
| 102 | GLI3\_TARGET\_GENES |  | 72 | 0.18 | 1.17 | 0.229 | 0.918 | 1.000 | 446 | tags=43%, list=32%, signal=60% |
| 103 | RCGCANGCGY\_NRF1\_Q6 |  | 93 | 0.17 | 1.17 | 0.243 | 0.921 | 1.000 | 742 | tags=66%, list=53%, signal=129% |
| 104 | E2F\_Q3 |  | 29 | 0.24 | 1.16 | 0.269 | 0.924 | 1.000 | 688 | tags=66%, list=49%, signal=125% |
| 105 | NPAT\_TARGET\_GENES |  | 18 | 0.28 | 1.16 | 0.263 | 0.925 | 1.000 | 153 | tags=28%, list=11%, signal=31% |
| 106 | ZNF362\_TARGET\_GENES |  | 46 | 0.21 | 1.16 | 0.275 | 0.917 | 1.000 | 580 | tags=54%, list=41%, signal=89% |
| 107 | YY1\_02 |  | 27 | 0.25 | 1.16 | 0.289 | 0.918 | 1.000 | 651 | tags=67%, list=46%, signal=121% |
| 108 | AP1\_Q2\_01 |  | 26 | 0.24 | 1.15 | 0.282 | 0.922 | 1.000 | 181 | tags=31%, list=13%, signal=35% |
| 109 | ZNF391\_TARGET\_GENES |  | 74 | 0.18 | 1.14 | 0.283 | 0.946 | 1.000 | 424 | tags=36%, list=30%, signal=49% |
| 110 | WHN\_B |  | 29 | 0.24 | 1.14 | 0.295 | 0.943 | 1.000 | 106 | tags=21%, list=8%, signal=22% |
| 111 | ZBTB24\_TARGET\_GENES |  | 161 | 0.15 | 1.14 | 0.257 | 0.942 | 1.000 | 422 | tags=35%, list=30%, signal=44% |
| 112 | SREBP\_Q3 |  | 21 | 0.26 | 1.14 | 0.304 | 0.935 | 1.000 | 71 | tags=19%, list=5%, signal=20% |
| 113 | ZIC1\_01 |  | 23 | 0.26 | 1.14 | 0.295 | 0.929 | 1.000 | 324 | tags=39%, list=23%, signal=50% |
| 114 | NKX2\_3\_TARGET\_GENES |  | 64 | 0.18 | 1.14 | 0.289 | 0.926 | 1.000 | 98 | tags=14%, list=7%, signal=14% |
| 115 | ZBED4\_TARGET\_GENES |  | 35 | 0.22 | 1.14 | 0.291 | 0.923 | 1.000 | 345 | tags=34%, list=24%, signal=44% |
| 116 | CEBPZ\_TARGET\_GENES |  | 125 | 0.15 | 1.13 | 0.263 | 0.916 | 1.000 | 646 | tags=56%, list=46%, signal=94% |
| 117 | PTF1BETA\_Q6 |  | 24 | 0.25 | 1.13 | 0.296 | 0.913 | 1.000 | 368 | tags=42%, list=26%, signal=55% |
| 118 | ZNF329\_TARGET\_GENES |  | 43 | 0.21 | 1.13 | 0.283 | 0.908 | 1.000 | 965 | tags=91%, list=68%, signal=278% |
| 119 | NKX2\_2\_TARGET\_GENES |  | 135 | 0.15 | 1.12 | 0.286 | 0.930 | 1.000 | 352 | tags=29%, list=25%, signal=35% |
| 120 | ALPHACP1\_01 |  | 22 | 0.26 | 1.12 | 0.314 | 0.923 | 1.000 | 83 | tags=18%, list=6%, signal=19% |
| 121 | AAANWWTGC\_UNKNOWN |  | 18 | 0.27 | 1.12 | 0.322 | 0.917 | 1.000 | 294 | tags=39%, list=21%, signal=48% |
| 122 | HSD17B8\_TARGET\_GENES |  | 31 | 0.23 | 1.12 | 0.308 | 0.916 | 1.000 | 576 | tags=55%, list=41%, signal=91% |
| 123 | RFX7\_TARGET\_GENES |  | 42 | 0.20 | 1.12 | 0.313 | 0.913 | 1.000 | 644 | tags=57%, list=46%, signal=102% |
| 124 | CEBP\_Q2\_01 |  | 34 | 0.22 | 1.11 | 0.305 | 0.921 | 1.000 | 376 | tags=41%, list=27%, signal=55% |
| 125 | ZNF7\_TARGET\_GENES |  | 80 | 0.17 | 1.11 | 0.308 | 0.919 | 1.000 | 402 | tags=34%, list=28%, signal=45% |
| 126 | SP1\_Q4\_01 |  | 16 | 0.28 | 1.10 | 0.341 | 0.943 | 1.000 | 132 | tags=25%, list=9%, signal=27% |
| 127 | ACTAYRNNNCCCR\_UNKNOWN |  | 49 | 0.19 | 1.10 | 0.336 | 0.938 | 1.000 | 547 | tags=51%, list=39%, signal=80% |
| 128 | AHRR\_TARGET\_GENES |  | 114 | 0.15 | 1.10 | 0.322 | 0.950 | 1.000 | 200 | tags=20%, list=14%, signal=22% |
| 129 | HMGB2\_TARGET\_GENES |  | 50 | 0.19 | 1.09 | 0.336 | 0.955 | 1.000 | 364 | tags=34%, list=26%, signal=44% |
| 130 | TSHZ1\_TARGET\_GENES |  | 16 | 0.28 | 1.09 | 0.368 | 0.953 | 1.000 | 131 | tags=25%, list=9%, signal=27% |
| 131 | CAGCTG\_AP4\_Q5 |  | 95 | 0.16 | 1.09 | 0.341 | 0.958 | 1.000 | 46 | tags=11%, list=3%, signal=10% |
| 132 | YGCGYRCGC\_UNKNOWN |  | 30 | 0.22 | 1.09 | 0.357 | 0.952 | 1.000 | 508 | tags=47%, list=36%, signal=71% |
| 133 | BANP\_TARGET\_GENES |  | 84 | 0.17 | 1.08 | 0.377 | 0.956 | 1.000 | 636 | tags=54%, list=45%, signal=92% |
| 134 | ATOH1\_TARGET\_GENES |  | 21 | 0.25 | 1.08 | 0.354 | 0.959 | 1.000 | 831 | tags=86%, list=59%, signal=205% |
| 135 | SPZ1\_01 |  | 20 | 0.26 | 1.08 | 0.350 | 0.955 | 1.000 | 24 | tags=15%, list=2%, signal=15% |
| 136 | ZF5\_B |  | 23 | 0.24 | 1.07 | 0.366 | 0.962 | 1.000 | 118 | tags=22%, list=8%, signal=23% |
| 137 | H1\_6\_TARGET\_GENES |  | 40 | 0.20 | 1.07 | 0.379 | 0.956 | 1.000 | 424 | tags=43%, list=30%, signal=59% |
| 138 | ZNF8\_TARGET\_GENES |  | 73 | 0.17 | 1.07 | 0.372 | 0.950 | 1.000 | 660 | tags=59%, list=47%, signal=105% |
| 139 | NFY\_Q6\_01 |  | 25 | 0.24 | 1.07 | 0.371 | 0.951 | 1.000 | 207 | tags=24%, list=15%, signal=28% |
| 140 | DLX2\_TARGET\_GENES |  | 34 | 0.20 | 1.06 | 0.382 | 0.967 | 1.000 | 413 | tags=38%, list=29%, signal=53% |
| 141 | NERF\_Q2 |  | 16 | 0.27 | 1.06 | 0.388 | 0.966 | 1.000 | 45 | tags=19%, list=3%, signal=19% |
| 142 | EGR\_Q6 |  | 15 | 0.28 | 1.06 | 0.376 | 0.963 | 1.000 | 234 | tags=33%, list=17%, signal=40% |
| 143 | SP1\_Q6 |  | 17 | 0.27 | 1.06 | 0.383 | 0.960 | 1.000 | 132 | tags=24%, list=9%, signal=26% |
| 144 | IRX2\_TARGET\_GENES |  | 20 | 0.25 | 1.06 | 0.358 | 0.959 | 1.000 | 292 | tags=40%, list=21%, signal=50% |
| 145 | TBX3\_TARGET\_GENES |  | 95 | 0.15 | 1.05 | 0.394 | 0.976 | 1.000 | 578 | tags=48%, list=41%, signal=76% |
| 146 | E2F\_Q6 |  | 34 | 0.21 | 1.05 | 0.403 | 0.969 | 1.000 | 688 | tags=65%, list=49%, signal=123% |
| 147 | LHX2\_TARGET\_GENES |  | 124 | 0.14 | 1.05 | 0.395 | 0.963 | 1.000 | 509 | tags=40%, list=36%, signal=58% |
| 148 | ZNF30\_TARGET\_GENES |  | 142 | 0.14 | 1.05 | 0.379 | 0.960 | 1.000 | 626 | tags=52%, list=44%, signal=84% |
| 149 | PU1\_Q6 |  | 18 | 0.25 | 1.05 | 0.405 | 0.961 | 1.000 | 140 | tags=22%, list=10%, signal=24% |
| 150 | E2F\_Q4 |  | 34 | 0.21 | 1.04 | 0.390 | 0.960 | 1.000 | 688 | tags=65%, list=49%, signal=123% |
| 151 | ZFP3\_TARGET\_GENES |  | 35 | 0.20 | 1.04 | 0.381 | 0.965 | 1.000 | 607 | tags=57%, list=43%, signal=98% |
| 152 | ZNF2\_TARGET\_GENES |  | 101 | 0.15 | 1.04 | 0.409 | 0.972 | 1.000 | 435 | tags=36%, list=31%, signal=48% |
| 153 | TCF4\_Q5 |  | 16 | 0.26 | 1.03 | 0.424 | 0.970 | 1.000 | 228 | tags=31%, list=16%, signal=37% |
| 154 | STAT1\_03 |  | 22 | 0.24 | 1.03 | 0.422 | 0.974 | 1.000 | 104 | tags=18%, list=7%, signal=19% |
| 155 | PIT1\_Q6 |  | 22 | 0.24 | 1.03 | 0.425 | 0.969 | 1.000 | 294 | tags=32%, list=21%, signal=40% |
| 156 | E2F1\_Q3 |  | 33 | 0.20 | 1.03 | 0.412 | 0.966 | 1.000 | 626 | tags=58%, list=44%, signal=101% |
| 157 | STTTCRNTTT\_IRF\_Q6 |  | 22 | 0.24 | 1.03 | 0.417 | 0.960 | 1.000 | 71 | tags=18%, list=5%, signal=19% |
| 158 | ASH1L\_TARGET\_GENES |  | 130 | 0.14 | 1.03 | 0.421 | 0.955 | 1.000 | 625 | tags=52%, list=44%, signal=84% |
| 159 | ELK1\_02 |  | 25 | 0.22 | 1.03 | 0.437 | 0.957 | 1.000 | 628 | tags=60%, list=45%, signal=106% |
| 160 | RRAGTTGT\_UNKNOWN |  | 29 | 0.21 | 1.02 | 0.397 | 0.958 | 1.000 | 488 | tags=45%, list=35%, signal=67% |
| 161 | TERF2\_TARGET\_GENES |  | 16 | 0.26 | 1.02 | 0.434 | 0.956 | 1.000 | 455 | tags=50%, list=32%, signal=73% |
| 162 | ZNF579\_TARGET\_GENES |  | 65 | 0.17 | 1.02 | 0.435 | 0.952 | 1.000 | 671 | tags=58%, list=48%, signal=106% |
| 163 | PRDM6\_TARGET\_GENES |  | 176 | 0.13 | 1.01 | 0.428 | 0.969 | 1.000 | 88 | tags=11%, list=6%, signal=10% |
| 164 | RLF\_TARGET\_GENES |  | 29 | 0.21 | 1.01 | 0.422 | 0.971 | 1.000 | 733 | tags=72%, list=52%, signal=148% |
| 165 | YKACATTT\_UNKNOWN |  | 22 | 0.23 | 1.00 | 0.436 | 0.987 | 1.000 | 83 | tags=18%, list=6%, signal=19% |
| 166 | MZF1\_TARGET\_GENES |  | 151 | 0.13 | 1.00 | 0.478 | 0.991 | 1.000 | 172 | tags=16%, list=12%, signal=16% |
| 167 | CATTGTYY\_SOX9\_B1 |  | 27 | 0.21 | 0.99 | 0.438 | 1.000 | 1.000 | 159 | tags=22%, list=11%, signal=25% |
| 168 | YY1\_Q6 |  | 30 | 0.20 | 0.99 | 0.459 | 1.000 | 1.000 | 651 | tags=60%, list=46%, signal=109% |
| 169 | STAT4\_01 |  | 25 | 0.22 | 0.99 | 0.481 | 1.000 | 1.000 | 71 | tags=16%, list=5%, signal=17% |
| 170 | KLF13\_TARGET\_GENES |  | 191 | 0.13 | 0.99 | 0.491 | 1.000 | 1.000 | 470 | tags=36%, list=33%, signal=46% |
| 171 | TGACCTTG\_SF1\_Q6 |  | 23 | 0.22 | 0.98 | 0.462 | 1.000 | 1.000 | 71 | tags=17%, list=5%, signal=18% |
| 172 | DYRK1A\_TARGET\_GENES |  | 78 | 0.15 | 0.98 | 0.482 | 1.000 | 1.000 | 459 | tags=37%, list=33%, signal=52% |
| 173 | ZNF740\_TARGET\_GENES |  | 139 | 0.13 | 0.97 | 0.495 | 1.000 | 1.000 | 345 | tags=26%, list=24%, signal=31% |
| 174 | GATA4\_Q3 |  | 16 | 0.25 | 0.97 | 0.473 | 1.000 | 1.000 | 150 | tags=25%, list=11%, signal=28% |
| 175 | JDP2\_TARGET\_GENES |  | 205 | 0.12 | 0.97 | 0.540 | 1.000 | 1.000 | 307 | tags=23%, list=22%, signal=26% |
| 176 | ZID\_01 |  | 23 | 0.22 | 0.97 | 0.491 | 1.000 | 1.000 | 132 | tags=17%, list=9%, signal=19% |
| 177 | SRSF7\_TARGET\_GENES |  | 16 | 0.24 | 0.97 | 0.503 | 1.000 | 1.000 | 337 | tags=38%, list=24%, signal=49% |
| 178 | FOXO4\_TARGET\_GENES |  | 31 | 0.20 | 0.96 | 0.519 | 1.000 | 1.000 | 725 | tags=71%, list=51%, signal=143% |
| 179 | SNAI1\_TARGET\_GENES |  | 22 | 0.22 | 0.96 | 0.491 | 1.000 | 1.000 | 344 | tags=36%, list=24%, signal=47% |
| 180 | NAB2\_TARGET\_GENES |  | 140 | 0.13 | 0.96 | 0.532 | 1.000 | 1.000 | 424 | tags=32%, list=30%, signal=41% |
| 181 | ZNF507\_TARGET\_GENES |  | 85 | 0.15 | 0.96 | 0.524 | 1.000 | 1.000 | 547 | tags=45%, list=39%, signal=69% |
| 182 | PITX1\_TARGET\_GENES |  | 30 | 0.20 | 0.96 | 0.512 | 1.000 | 1.000 | 535 | tags=50%, list=38%, signal=79% |
| 183 | AREB6\_04 |  | 21 | 0.22 | 0.96 | 0.505 | 1.000 | 1.000 | 132 | tags=19%, list=9%, signal=21% |
| 184 | AML\_Q6 |  | 22 | 0.22 | 0.96 | 0.510 | 1.000 | 1.000 | 54 | tags=14%, list=4%, signal=14% |
| 185 | HAND1E47\_01 |  | 26 | 0.20 | 0.94 | 0.526 | 1.000 | 1.000 | 16 | tags=12%, list=1%, signal=11% |
| 186 | ZNF320\_TARGET\_GENES |  | 119 | 0.13 | 0.94 | 0.548 | 1.000 | 1.000 | 592 | tags=46%, list=42%, signal=73% |
| 187 | GATAAGR\_GATA\_C |  | 15 | 0.25 | 0.94 | 0.504 | 1.000 | 1.000 | 83 | tags=20%, list=6%, signal=21% |
| 188 | NCOA2\_TARGET\_GENES |  | 40 | 0.18 | 0.94 | 0.534 | 1.000 | 1.000 | 267 | tags=25%, list=19%, signal=30% |
| 189 | HNRNPUL1\_TARGET\_GENES |  | 144 | 0.13 | 0.94 | 0.562 | 1.000 | 1.000 | 193 | tags=16%, list=14%, signal=17% |
| 190 | ERR1\_Q2 |  | 23 | 0.21 | 0.94 | 0.514 | 1.000 | 1.000 | 240 | tags=26%, list=17%, signal=31% |
| 191 | ZNF274\_TARGET\_GENES |  | 79 | 0.15 | 0.94 | 0.554 | 1.000 | 1.000 | 427 | tags=38%, list=30%, signal=51% |
| 192 | KAT5\_TARGET\_GENES |  | 91 | 0.14 | 0.93 | 0.576 | 1.000 | 1.000 | 706 | tags=57%, list=50%, signal=107% |
| 193 | AP2\_Q3 |  | 19 | 0.22 | 0.93 | 0.533 | 1.000 | 1.000 | 649 | tags=63%, list=46%, signal=115% |
| 194 | ZNF596\_TARGET\_GENES |  | 50 | 0.16 | 0.93 | 0.560 | 1.000 | 1.000 | 314 | tags=30%, list=22%, signal=37% |
| 195 | KDM5D\_TARGET\_GENES |  | 172 | 0.12 | 0.93 | 0.592 | 1.000 | 1.000 | 759 | tags=63%, list=54%, signal=119% |
| 196 | CBX7\_TARGET\_GENES |  | 72 | 0.15 | 0.93 | 0.577 | 1.000 | 1.000 | 501 | tags=43%, list=36%, signal=63% |
| 197 | ZNF843\_TARGET\_GENES |  | 80 | 0.14 | 0.93 | 0.571 | 1.000 | 1.000 | 372 | tags=31%, list=26%, signal=40% |
| 198 | CREB\_Q2\_01 |  | 20 | 0.22 | 0.92 | 0.565 | 1.000 | 1.000 | 148 | tags=20%, list=10%, signal=22% |
| 199 | ZNF592\_TARGET\_GENES |  | 161 | 0.12 | 0.92 | 0.588 | 1.000 | 1.000 | 282 | tags=22%, list=20%, signal=25% |
| 200 | ID2\_TARGET\_GENES |  | 102 | 0.13 | 0.92 | 0.577 | 1.000 | 1.000 | 487 | tags=39%, list=35%, signal=56% |
| 201 | PAX6\_TARGET\_GENES |  | 71 | 0.14 | 0.91 | 0.599 | 1.000 | 1.000 | 146 | tags=15%, list=10%, signal=16% |
| 202 | PAX4\_03 |  | 16 | 0.23 | 0.91 | 0.569 | 1.000 | 1.000 | 71 | tags=19%, list=5%, signal=20% |
| 203 | ZNF410\_TARGET\_GENES |  | 59 | 0.15 | 0.91 | 0.566 | 1.000 | 1.000 | 544 | tags=49%, list=39%, signal=77% |
| 204 | TTF1\_Q6 |  | 20 | 0.21 | 0.91 | 0.591 | 1.000 | 1.000 | 160 | tags=20%, list=11%, signal=22% |
| 205 | ZSCAN21\_TARGET\_GENES |  | 48 | 0.16 | 0.91 | 0.592 | 1.000 | 1.000 | 145 | tags=17%, list=10%, signal=18% |
| 206 | CIZ\_01 |  | 24 | 0.20 | 0.90 | 0.564 | 1.000 | 1.000 | 29 | tags=13%, list=2%, signal=13% |
| 207 | LYF1\_01 |  | 22 | 0.21 | 0.90 | 0.569 | 1.000 | 1.000 | 132 | tags=18%, list=9%, signal=20% |
| 208 | CSHL1\_TARGET\_GENES |  | 24 | 0.20 | 0.90 | 0.581 | 1.000 | 1.000 | 324 | tags=33%, list=23%, signal=43% |
| 209 | GTF2A2\_TARGET\_GENES |  | 56 | 0.15 | 0.90 | 0.595 | 1.000 | 1.000 | 238 | tags=20%, list=17%, signal=23% |
| 210 | CC2D1A\_TARGET\_GENES |  | 121 | 0.12 | 0.90 | 0.647 | 1.000 | 1.000 | 603 | tags=50%, list=43%, signal=79% |
| 211 | CDX2\_Q5 |  | 19 | 0.22 | 0.90 | 0.574 | 1.000 | 1.000 | 216 | tags=26%, list=15%, signal=31% |
| 212 | STAT5A\_03 |  | 20 | 0.21 | 0.90 | 0.597 | 1.000 | 1.000 | 132 | tags=20%, list=9%, signal=22% |
| 213 | TCANNTGAY\_SREBP1\_01 |  | 49 | 0.16 | 0.90 | 0.596 | 1.000 | 1.000 | 589 | tags=51%, list=42%, signal=85% |
| 214 | ELF5\_TARGET\_GENES |  | 40 | 0.17 | 0.89 | 0.602 | 1.000 | 1.000 | 356 | tags=35%, list=25%, signal=45% |
| 215 | TGATTTRY\_GFI1\_01 |  | 16 | 0.23 | 0.89 | 0.575 | 1.000 | 1.000 | 294 | tags=38%, list=21%, signal=47% |
| 216 | VDR\_Q3 |  | 19 | 0.21 | 0.89 | 0.601 | 1.000 | 1.000 | 237 | tags=26%, list=17%, signal=31% |
| 217 | TGACCTY\_ERR1\_Q2 |  | 78 | 0.14 | 0.89 | 0.632 | 1.000 | 1.000 | 82 | tags=12%, list=6%, signal=12% |
| 218 | HSF2\_TARGET\_GENES |  | 31 | 0.18 | 0.89 | 0.612 | 1.000 | 1.000 | 537 | tags=48%, list=38%, signal=76% |
| 219 | PCGF1\_TARGET\_GENES |  | 49 | 0.16 | 0.89 | 0.644 | 1.000 | 1.000 | 495 | tags=43%, list=35%, signal=64% |
| 220 | PRKDC\_TARGET\_GENES |  | 64 | 0.15 | 0.88 | 0.625 | 1.000 | 1.000 | 506 | tags=44%, list=36%, signal=65% |
| 221 | E2F\_03 |  | 28 | 0.18 | 0.88 | 0.598 | 1.000 | 1.000 | 688 | tags=64%, list=49%, signal=123% |
| 222 | ISRE\_01 |  | 28 | 0.18 | 0.88 | 0.613 | 1.000 | 1.000 | 324 | tags=32%, list=23%, signal=41% |
| 223 | PITX2\_Q2 |  | 24 | 0.20 | 0.88 | 0.629 | 1.000 | 1.000 | 54 | tags=13%, list=4%, signal=13% |
| 224 | TEAD2\_TARGET\_GENES |  | 143 | 0.12 | 0.88 | 0.672 | 1.000 | 1.000 | 244 | tags=20%, list=17%, signal=21% |
| 225 | TEF\_Q6 |  | 24 | 0.20 | 0.87 | 0.622 | 1.000 | 1.000 | 54 | tags=13%, list=4%, signal=13% |
| 226 | ZNF282\_TARGET\_GENES |  | 88 | 0.13 | 0.87 | 0.638 | 1.000 | 1.000 | 522 | tags=42%, list=37%, signal=63% |
| 227 | SF1\_Q6 |  | 25 | 0.19 | 0.87 | 0.633 | 1.000 | 1.000 | 39 | tags=12%, list=3%, signal=12% |
| 228 | TITF1\_Q3 |  | 21 | 0.20 | 0.87 | 0.630 | 1.000 | 1.000 | 198 | tags=24%, list=14%, signal=27% |
| 229 | PAX7\_TARGET\_GENES |  | 49 | 0.15 | 0.86 | 0.657 | 1.000 | 1.000 | 347 | tags=31%, list=25%, signal=39% |
| 230 | LCORL\_TARGET\_GENES |  | 37 | 0.17 | 0.86 | 0.638 | 1.000 | 1.000 | 276 | tags=27%, list=20%, signal=33% |
| 231 | ZC3H11A\_TARGET\_GENES |  | 85 | 0.13 | 0.86 | 0.671 | 1.000 | 1.000 | 704 | tags=60%, list=50%, signal=113% |
| 232 | RAG1\_TARGET\_GENES |  | 111 | 0.12 | 0.85 | 0.699 | 1.000 | 1.000 | 248 | tags=20%, list=18%, signal=22% |
| 233 | YATTNATC\_UNKNOWN |  | 33 | 0.17 | 0.85 | 0.633 | 1.000 | 1.000 | 63 | tags=12%, list=4%, signal=12% |
| 234 | TET1\_SPLICE\_VARIANT\_VP\_DE456\_TARGET\_GENES |  | 25 | 0.19 | 0.85 | 0.667 | 1.000 | 1.000 | 50 | tags=12%, list=4%, signal=12% |
| 235 | COREBINDINGFACTOR\_Q6 |  | 17 | 0.21 | 0.85 | 0.642 | 1.000 | 1.000 | 29 | tags=12%, list=2%, signal=12% |
| 236 | SGCGSSAAA\_E2F1DP2\_01 |  | 22 | 0.19 | 0.85 | 0.652 | 1.000 | 1.000 | 612 | tags=55%, list=43%, signal=95% |
| 237 | WTTGKCTG\_UNKNOWN |  | 43 | 0.16 | 0.85 | 0.663 | 1.000 | 1.000 | 257 | tags=23%, list=18%, signal=28% |
| 238 | IRF7\_01 |  | 24 | 0.19 | 0.85 | 0.643 | 1.000 | 1.000 | 258 | tags=29%, list=18%, signal=35% |
| 239 | RORA\_TARGET\_GENES |  | 21 | 0.20 | 0.84 | 0.646 | 1.000 | 1.000 | 644 | tags=67%, list=46%, signal=121% |
| 240 | E2F1DP1RB\_01 |  | 36 | 0.16 | 0.84 | 0.658 | 1.000 | 1.000 | 1077 | tags=94%, list=76%, signal=389% |
| 241 | ZFP69B\_TARGET\_GENES |  | 174 | 0.11 | 0.84 | 0.733 | 1.000 | 1.000 | 481 | tags=36%, list=34%, signal=47% |
| 242 | PAX3\_TARGET\_GENES |  | 113 | 0.12 | 0.84 | 0.727 | 1.000 | 1.000 | 287 | tags=23%, list=20%, signal=27% |
| 243 | MSX1\_TARGET\_GENES |  | 16 | 0.22 | 0.84 | 0.643 | 1.000 | 1.000 | 29 | tags=13%, list=2%, signal=13% |
| 244 | FOXE1\_TARGET\_GENES |  | 83 | 0.13 | 0.83 | 0.703 | 1.000 | 1.000 | 644 | tags=54%, list=46%, signal=94% |
| 245 | ZSCAN30\_TARGET\_GENES |  | 159 | 0.11 | 0.83 | 0.733 | 1.000 | 1.000 | 192 | tags=16%, list=14%, signal=16% |
| 246 | MIF1\_01 |  | 22 | 0.19 | 0.83 | 0.671 | 1.000 | 1.000 | 71 | tags=14%, list=5%, signal=14% |
| 247 | ATF6\_TARGET\_GENES |  | 94 | 0.12 | 0.83 | 0.732 | 1.000 | 1.000 | 528 | tags=40%, list=37%, signal=60% |
| 248 | SNIP1\_TARGET\_GENES |  | 75 | 0.13 | 0.83 | 0.711 | 1.000 | 1.000 | 676 | tags=55%, list=48%, signal=99% |
| 249 | ZNF490\_TARGET\_GENES |  | 37 | 0.16 | 0.83 | 0.680 | 0.998 | 1.000 | 527 | tags=49%, list=37%, signal=76% |
| 250 | STAT5A\_01 |  | 22 | 0.19 | 0.83 | 0.692 | 0.995 | 1.000 | 154 | tags=18%, list=11%, signal=20% |
| 251 | NR1I2\_TARGET\_GENES |  | 44 | 0.15 | 0.83 | 0.688 | 0.994 | 1.000 | 839 | tags=77%, list=59%, signal=185% |
| 252 | GUCY1B1\_TARGET\_GENES |  | 66 | 0.13 | 0.82 | 0.702 | 0.999 | 1.000 | 78 | tags=11%, list=6%, signal=11% |
| 253 | ZNF140\_TARGET\_GENES |  | 54 | 0.14 | 0.82 | 0.705 | 0.997 | 1.000 | 202 | tags=19%, list=14%, signal=21% |
| 254 | MZF1\_01 |  | 19 | 0.20 | 0.82 | 0.698 | 0.997 | 1.000 | 71 | tags=16%, list=5%, signal=16% |
| 255 | ZNF623\_TARGET\_GENES |  | 23 | 0.18 | 0.82 | 0.702 | 0.999 | 1.000 | 675 | tags=61%, list=48%, signal=115% |
| 256 | ZFP91\_TARGET\_GENES |  | 106 | 0.12 | 0.82 | 0.735 | 0.997 | 1.000 | 394 | tags=30%, list=28%, signal=39% |
| 257 | WTGAAAT\_UNKNOWN |  | 47 | 0.14 | 0.81 | 0.707 | 1.000 | 1.000 | 366 | tags=32%, list=26%, signal=42% |
| 258 | CDC5\_01 |  | 24 | 0.18 | 0.81 | 0.697 | 0.998 | 1.000 | 118 | tags=17%, list=8%, signal=18% |
| 259 | SALL4\_TARGET\_GENES |  | 184 | 0.10 | 0.81 | 0.790 | 0.995 | 1.000 | 497 | tags=38%, list=35%, signal=50% |
| 260 | E2F1\_Q3\_01 |  | 27 | 0.17 | 0.81 | 0.701 | 0.997 | 1.000 | 5 | tags=7%, list=0%, signal=7% |
| 261 | E2F5\_TARGET\_GENES |  | 115 | 0.11 | 0.80 | 0.757 | 1.000 | 1.000 | 553 | tags=43%, list=39%, signal=66% |
| 262 | ZNF331\_TARGET\_GENES |  | 37 | 0.15 | 0.80 | 0.710 | 1.000 | 1.000 | 520 | tags=46%, list=37%, signal=71% |
| 263 | SS18\_SSX1\_FUSION\_TARGET\_GENES |  | 33 | 0.16 | 0.80 | 0.739 | 1.000 | 1.000 | 148 | tags=18%, list=10%, signal=20% |
| 264 | CETS1P54\_01 |  | 27 | 0.17 | 0.80 | 0.739 | 1.000 | 1.000 | 372 | tags=33%, list=26%, signal=44% |
| 265 | E2F\_Q6\_01 |  | 29 | 0.16 | 0.79 | 0.730 | 1.000 | 1.000 | 688 | tags=59%, list=49%, signal=112% |
| 266 | HMGIY\_Q6 |  | 15 | 0.21 | 0.79 | 0.726 | 1.000 | 1.000 | 34 | tags=13%, list=2%, signal=14% |
| 267 | PAX4\_04 |  | 20 | 0.18 | 0.79 | 0.715 | 1.000 | 1.000 | 165 | tags=20%, list=12%, signal=22% |
| 268 | E2F4DP1\_01 |  | 33 | 0.16 | 0.78 | 0.733 | 1.000 | 1.000 | 633 | tags=52%, list=45%, signal=91% |
| 269 | GTF2E2\_TARGET\_GENES |  | 42 | 0.15 | 0.78 | 0.772 | 1.000 | 1.000 | 87 | tags=12%, list=6%, signal=12% |
| 270 | CDC5L\_TARGET\_GENES |  | 17 | 0.20 | 0.78 | 0.721 | 1.000 | 1.000 | 628 | tags=65%, list=45%, signal=115% |
| 271 | ATF4\_Q2 |  | 18 | 0.19 | 0.78 | 0.725 | 1.000 | 1.000 | 26 | tags=11%, list=2%, signal=11% |
| 272 | NOTCH3\_TARGET\_GENES |  | 121 | 0.11 | 0.78 | 0.809 | 0.999 | 1.000 | 512 | tags=40%, list=36%, signal=57% |
| 273 | ZMYND8\_TARGET\_GENES |  | 188 | 0.10 | 0.78 | 0.820 | 0.996 | 1.000 | 65 | tags=8%, list=5%, signal=7% |
| 274 | E2F1\_Q6 |  | 30 | 0.16 | 0.78 | 0.729 | 0.994 | 1.000 | 633 | tags=53%, list=45%, signal=95% |
| 275 | ZNF41\_TARGET\_GENES |  | 16 | 0.20 | 0.78 | 0.730 | 0.995 | 1.000 | 45 | tags=13%, list=3%, signal=13% |
| 276 | ZNF134\_TARGET\_GENES |  | 86 | 0.12 | 0.78 | 0.779 | 0.993 | 1.000 | 522 | tags=42%, list=37%, signal=62% |
| 277 | SETD7\_TARGET\_GENES |  | 95 | 0.11 | 0.77 | 0.801 | 0.994 | 1.000 | 846 | tags=71%, list=60%, signal=164% |
| 278 | KLF17\_TARGET\_GENES |  | 174 | 0.10 | 0.77 | 0.825 | 0.996 | 1.000 | 481 | tags=36%, list=34%, signal=48% |
| 279 | ATRX\_TARGET\_GENES |  | 127 | 0.11 | 0.77 | 0.828 | 0.993 | 1.000 | 493 | tags=39%, list=35%, signal=55% |
| 280 | E4F1\_Q6 |  | 32 | 0.15 | 0.76 | 0.784 | 0.999 | 1.000 | 1039 | tags=91%, list=74%, signal=336% |
| 281 | CTNNB1\_TARGET\_GENES |  | 42 | 0.14 | 0.76 | 0.782 | 1.000 | 1.000 | 577 | tags=50%, list=41%, signal=82% |
| 282 | MXD1\_TARGET\_GENES |  | 62 | 0.12 | 0.75 | 0.815 | 1.000 | 1.000 | 752 | tags=65%, list=53%, signal=132% |
| 283 | ZNF528\_TARGET\_GENES |  | 66 | 0.12 | 0.75 | 0.832 | 1.000 | 1.000 | 89 | tags=11%, list=6%, signal=11% |
| 284 | ZNF610\_TARGET\_GENES |  | 79 | 0.11 | 0.75 | 0.813 | 1.000 | 1.000 | 469 | tags=37%, list=33%, signal=52% |
| 285 | HMG20B\_TARGET\_GENES |  | 163 | 0.10 | 0.75 | 0.851 | 1.000 | 1.000 | 202 | tags=15%, list=14%, signal=16% |
| 286 | NFE2\_01 |  | 21 | 0.18 | 0.75 | 0.778 | 1.000 | 1.000 | 240 | tags=24%, list=17%, signal=28% |
| 287 | MORC2\_TARGET\_GENES |  | 219 | 0.09 | 0.74 | 0.879 | 1.000 | 1.000 | 740 | tags=58%, list=52%, signal=104% |
| 288 | ZNF223\_TARGET\_GENES |  | 19 | 0.18 | 0.74 | 0.762 | 1.000 | 1.000 | 724 | tags=74%, list=51%, signal=149% |
| 289 | FAC1\_01 |  | 21 | 0.17 | 0.73 | 0.798 | 1.000 | 1.000 | 302 | tags=29%, list=21%, signal=36% |
| 290 | TFEB\_TARGET\_GENES |  | 143 | 0.10 | 0.73 | 0.872 | 1.000 | 1.000 | 416 | tags=31%, list=29%, signal=40% |
| 291 | ZNF549\_TARGET\_GENES |  | 102 | 0.11 | 0.73 | 0.838 | 1.000 | 1.000 | 155 | tags=14%, list=11%, signal=14% |
| 292 | E2F4DP2\_01 |  | 31 | 0.15 | 0.73 | 0.801 | 1.000 | 1.000 | 1077 | tags=94%, list=76%, signal=387% |
| 293 | SP1\_Q6\_01 |  | 18 | 0.18 | 0.73 | 0.831 | 1.000 | 1.000 | 132 | tags=17%, list=9%, signal=18% |
| 294 | E2F1DP2\_01 |  | 31 | 0.15 | 0.73 | 0.832 | 1.000 | 1.000 | 1077 | tags=94%, list=76%, signal=387% |
| 295 | FOXD2\_TARGET\_GENES |  | 108 | 0.10 | 0.73 | 0.857 | 1.000 | 1.000 | 389 | tags=29%, list=28%, signal=37% |
| 296 | HOXA10\_TARGET\_GENES |  | 20 | 0.17 | 0.73 | 0.798 | 1.000 | 1.000 | 544 | tags=50%, list=39%, signal=80% |
| 297 | HDAC4\_TARGET\_GENES |  | 54 | 0.13 | 0.72 | 0.850 | 0.999 | 1.000 | 542 | tags=43%, list=38%, signal=67% |
| 298 | E2F1DP1\_01 |  | 31 | 0.15 | 0.72 | 0.813 | 1.000 | 1.000 | 1077 | tags=94%, list=76%, signal=387% |
| 299 | FXR1\_TARGET\_GENES |  | 110 | 0.10 | 0.72 | 0.873 | 0.999 | 1.000 | 732 | tags=60%, list=52%, signal=115% |
| 300 | SYATTGTG\_UNKNOWN |  | 17 | 0.18 | 0.72 | 0.811 | 0.999 | 1.000 | 118 | tags=18%, list=8%, signal=19% |
| 301 | ZFHX3\_TARGET\_GENES |  | 136 | 0.09 | 0.71 | 0.889 | 1.000 | 1.000 | 285 | tags=21%, list=20%, signal=24% |
| 302 | TCCATTKW\_UNKNOWN |  | 25 | 0.16 | 0.71 | 0.837 | 1.000 | 1.000 | 159 | tags=16%, list=11%, signal=18% |
| 303 | ZNF224\_TARGET\_GENES |  | 121 | 0.10 | 0.71 | 0.900 | 0.997 | 1.000 | 729 | tags=58%, list=52%, signal=109% |
| 304 | SETBP1\_TARGET\_GENES |  | 16 | 0.18 | 0.71 | 0.821 | 0.994 | 1.000 | 166 | tags=19%, list=12%, signal=21% |
| 305 | E2F\_02 |  | 31 | 0.15 | 0.71 | 0.842 | 0.996 | 1.000 | 1077 | tags=94%, list=76%, signal=387% |
| 306 | FEV\_TARGET\_GENES |  | 141 | 0.10 | 0.70 | 0.888 | 0.997 | 1.000 | 551 | tags=42%, list=39%, signal=62% |
| 307 | HFH1\_01 |  | 24 | 0.15 | 0.70 | 0.810 | 0.994 | 1.000 | 23 | tags=8%, list=2%, signal=8% |
| 308 | AR\_Q2 |  | 17 | 0.17 | 0.70 | 0.814 | 0.991 | 1.000 | 63 | tags=12%, list=4%, signal=12% |
| 309 | RREB1\_01 |  | 15 | 0.19 | 0.70 | 0.846 | 0.989 | 1.000 | 257 | tags=27%, list=18%, signal=32% |
| 310 | ZNF101\_TARGET\_GENES |  | 40 | 0.13 | 0.70 | 0.857 | 0.988 | 1.000 | 686 | tags=58%, list=49%, signal=109% |
| 311 | SREBP1\_01 |  | 20 | 0.17 | 0.69 | 0.832 | 0.991 | 1.000 | 487 | tags=45%, list=35%, signal=68% |
| 312 | ZNF830\_TARGET\_GENES |  | 28 | 0.15 | 0.69 | 0.831 | 0.991 | 1.000 | 729 | tags=64%, list=52%, signal=130% |
| 313 | GTGACGY\_E4F1\_Q6 |  | 61 | 0.11 | 0.68 | 0.865 | 0.999 | 1.000 | 25 | tags=7%, list=2%, signal=6% |
| 314 | ZNF184\_TARGET\_GENES |  | 123 | 0.09 | 0.68 | 0.911 | 1.000 | 1.000 | 644 | tags=52%, list=46%, signal=87% |
| 315 | ZIC2\_01 |  | 16 | 0.17 | 0.67 | 0.874 | 1.000 | 1.000 | 390 | tags=31%, list=28%, signal=43% |
| 316 | RBM15\_TARGET\_GENES |  | 16 | 0.17 | 0.66 | 0.862 | 1.000 | 1.000 | 716 | tags=69%, list=51%, signal=138% |
| 317 | NFY\_C |  | 25 | 0.15 | 0.66 | 0.854 | 1.000 | 1.000 | 74 | tags=12%, list=5%, signal=12% |
| 318 | POU3F2\_02 |  | 20 | 0.16 | 0.65 | 0.871 | 1.000 | 1.000 | 567 | tags=50%, list=40%, signal=82% |
| 319 | CIC\_TARGET\_GENES |  | 60 | 0.11 | 0.65 | 0.895 | 1.000 | 1.000 | 423 | tags=33%, list=30%, signal=46% |
| 320 | ZIM3\_TARGET\_GENES |  | 84 | 0.10 | 0.64 | 0.922 | 1.000 | 1.000 | 74 | tags=8%, list=5%, signal=8% |
| 321 | NFXL1\_TARGET\_GENES |  | 28 | 0.14 | 0.64 | 0.907 | 1.000 | 1.000 | 198 | tags=18%, list=14%, signal=20% |
| 322 | ZNF395\_TARGET\_GENES |  | 89 | 0.09 | 0.64 | 0.912 | 1.000 | 1.000 | 734 | tags=60%, list=52%, signal=116% |
| 323 | DLX6\_TARGET\_GENES |  | 66 | 0.10 | 0.63 | 0.922 | 1.000 | 1.000 | 185 | tags=15%, list=13%, signal=17% |
| 324 | SATB1\_TARGET\_GENES |  | 16 | 0.16 | 0.63 | 0.916 | 1.000 | 1.000 | 271 | tags=25%, list=19%, signal=31% |
| 325 | E2F2\_TARGET\_GENES |  | 126 | 0.09 | 0.63 | 0.956 | 1.000 | 1.000 | 609 | tags=45%, list=43%, signal=72% |
| 326 | EBNA1BP2\_TARGET\_GENES |  | 30 | 0.13 | 0.63 | 0.893 | 1.000 | 1.000 | 12 | tags=7%, list=1%, signal=7% |
| 327 | CREB\_01 |  | 23 | 0.14 | 0.63 | 0.901 | 1.000 | 1.000 | 165 | tags=17%, list=12%, signal=19% |
| 328 | PCBP2\_TARGET\_GENES |  | 63 | 0.10 | 0.63 | 0.911 | 1.000 | 1.000 | 620 | tags=49%, list=44%, signal=84% |
| 329 | HNF1\_C |  | 19 | 0.15 | 0.63 | 0.902 | 0.999 | 1.000 | 1200 | tags=100%, list=85%, signal=660% |
| 330 | SUMO1\_TARGET\_GENES |  | 84 | 0.09 | 0.63 | 0.939 | 0.998 | 1.000 | 215 | tags=15%, list=15%, signal=17% |
| 331 | ID1\_TARGET\_GENES |  | 112 | 0.09 | 0.62 | 0.952 | 0.997 | 1.000 | 540 | tags=39%, list=38%, signal=59% |
| 332 | CREB\_Q3 |  | 25 | 0.13 | 0.62 | 0.913 | 0.999 | 1.000 | 695 | tags=60%, list=49%, signal=116% |
| 333 | CACGTG\_MYC\_Q2 |  | 117 | 0.09 | 0.61 | 0.962 | 1.000 | 1.000 | 223 | tags=16%, list=16%, signal=18% |
| 334 | ZNF816\_TARGET\_GENES |  | 46 | 0.11 | 0.61 | 0.941 | 1.000 | 1.000 | 644 | tags=52%, list=46%, signal=93% |
| 335 | RBM34\_TARGET\_GENES |  | 82 | 0.09 | 0.60 | 0.957 | 1.000 | 1.000 | 502 | tags=37%, list=36%, signal=53% |
| 336 | ARNT2\_TARGET\_GENES |  | 66 | 0.10 | 0.60 | 0.947 | 0.999 | 1.000 | 209 | tags=15%, list=15%, signal=17% |
| 337 | ER\_Q6\_02 |  | 21 | 0.14 | 0.60 | 0.923 | 0.998 | 1.000 | 1033 | tags=90%, list=73%, signal=333% |
| 338 | SRSF4\_TARGET\_GENES |  | 65 | 0.09 | 0.58 | 0.952 | 1.000 | 1.000 | 655 | tags=51%, list=46%, signal=90% |
| 339 | MYC\_Q2 |  | 19 | 0.14 | 0.58 | 0.936 | 1.000 | 1.000 | 529 | tags=47%, list=37%, signal=75% |
| 340 | SP3\_Q3 |  | 22 | 0.13 | 0.58 | 0.932 | 1.000 | 1.000 | 324 | tags=27%, list=23%, signal=35% |
| 341 | USF2\_Q6 |  | 22 | 0.13 | 0.58 | 0.935 | 1.000 | 1.000 | 529 | tags=45%, list=37%, signal=72% |
| 342 | CUX1\_TARGET\_GENES |  | 70 | 0.09 | 0.57 | 0.955 | 0.999 | 1.000 | 286 | tags=23%, list=20%, signal=27% |
| 343 | BARX1\_TARGET\_GENES |  | 106 | 0.08 | 0.56 | 0.978 | 1.000 | 1.000 | 544 | tags=41%, list=39%, signal=61% |
| 344 | KLF7\_TARGET\_GENES |  | 88 | 0.09 | 0.56 | 0.970 | 0.999 | 1.000 | 467 | tags=34%, list=33%, signal=48% |
| 345 | NFY\_01 |  | 18 | 0.13 | 0.56 | 0.943 | 0.999 | 1.000 | 3 | tags=6%, list=0%, signal=5% |
| 346 | OCT1\_06 |  | 21 | 0.13 | 0.56 | 0.962 | 0.997 | 1.000 | 551 | tags=43%, list=39%, signal=69% |
| 347 | ATF\_01 |  | 22 | 0.13 | 0.56 | 0.954 | 0.995 | 1.000 | 630 | tags=55%, list=45%, signal=97% |
| 348 | CREBP1CJUN\_01 |  | 25 | 0.12 | 0.55 | 0.961 | 0.992 | 1.000 | 656 | tags=56%, list=46%, signal=103% |
| 349 | HHEX\_TARGET\_GENES |  | 101 | 0.08 | 0.55 | 0.975 | 0.992 | 1.000 | 310 | tags=22%, list=22%, signal=26% |
| 350 | ZNF34\_TARGET\_GENES |  | 26 | 0.12 | 0.54 | 0.970 | 0.993 | 1.000 | 1044 | tags=88%, list=74%, signal=334% |
| 351 | ZNF197\_TARGET\_GENES |  | 60 | 0.09 | 0.54 | 0.974 | 0.991 | 1.000 | 650 | tags=50%, list=46%, signal=89% |
| 352 | HNRNPH1\_TARGET\_GENES |  | 169 | 0.07 | 0.53 | 0.993 | 0.992 | 1.000 | 862 | tags=69%, list=61%, signal=157% |
| 353 | CREBP1\_Q2 |  | 20 | 0.12 | 0.53 | 0.989 | 0.990 | 1.000 | 243 | tags=20%, list=17%, signal=24% |
| 354 | GCM\_Q2 |  | 19 | 0.12 | 0.52 | 0.968 | 0.991 | 1.000 | 77 | tags=11%, list=5%, signal=11% |
| 355 | CBX5\_TARGET\_GENES |  | 50 | 0.09 | 0.52 | 0.991 | 0.988 | 1.000 | 80 | tags=8%, list=6%, signal=8% |
| 356 | ZNF558\_TARGET\_GENES |  | 18 | 0.12 | 0.50 | 0.981 | 0.990 | 1.000 | 1240 | tags=100%, list=88%, signal=815% |
| 357 | ZNF322\_TARGET\_GENES |  | 60 | 0.08 | 0.50 | 0.981 | 0.988 | 1.000 | 1141 | tags=93%, list=81%, signal=467% |
| 358 | COMP1\_01 |  | 15 | 0.13 | 0.48 | 0.974 | 0.990 | 1.000 | 544 | tags=47%, list=39%, signal=75% |
| 359 | SIX1\_TARGET\_GENES |  | 22 | 0.10 | 0.45 | 0.995 | 0.993 | 1.000 | 149 | tags=14%, list=11%, signal=15% |
Table: Gene sets enriched in phenotype **WT (3 samples) **[plain text format]****
